# Genome-Wide Association Study Reveals Marker Trait Associations (MTA) for Waterlogging-Triggered Adventitious Roots and Aerenchyma Formation in Barley

**DOI:** 10.3390/ijms23063341

**Published:** 2022-03-19

**Authors:** S. M. Nuruzzaman Manik, Md Quamruzzaman, Chenchen Zhao, Peter Johnson, Ian Hunt, Sergey Shabala, Meixue Zhou

**Affiliations:** 1 Tasmanian Institute of Agriculture, University of Tasmania, Launceston, TAS 7250, Australia; smnuruzzaman.manik@utas.edu.au (S.M.N.M.); md.quamruzzaman@utas.edu.au (M.Q.); chenchen.zhao@utas.edu.au (C.Z.); p.g.johnson@utas.edu.au (P.J.); ian.hunt@utas.edu.au (I.H.); sergey.shabala@utas.edu.au (S.S.); 2 International Research Centre for Environmental Membrane Biology, Foshan University, Foshan 528000, China

**Keywords:** hypoxia, stress, aerenchyma formation, adventitious roots, *Hordeum vulgare*

## Abstract

Waterlogging is an environmental stress, which severely affects barley growth and development. Limited availability of oxygen in the root zone negatively affects the metabolism of the whole plant. Adventitious roots (AR) and root cortical aerenchyma (RCA) formation are the most important adaptive traits that contribute to a plant’s ability to survive in waterlogged soil conditions. This study used a genome-wide association (GWAS) approach using 18,132 single nucleotide polymorphisms (SNPs) in a panel of 697 barley genotypes to reveal marker trait associations (MTA) conferring the above adaptive traits. Experiments were conducted over two consecutive years in tanks filled with soil and then validated in field experiments. GWAS analysis was conducted using general linear models (GLM), mixed linear models (MLM), and fixed and random model circulating probability unification models (FarmCPU model), with the FarmCPU showing to be the best suited model. Six and five significant (approximately −log_10_ (*p*) ≥ 5.5) MTA were identified for AR and RCA formation under waterlogged conditions, respectively. The highest −log_10_ (*p*) MTA for adventitious root and aerenchyma formation were approximately 9 and 8 on chromosome 2H and 4H, respectively. The combination of different MTA showed to be more effective in forming RCA and producing more AR under waterlogging stress. Genes from major facilitator superfamily (*MFS*) transporter and leucine-rich repeat (*LRR*) families for AR formation, and ethylene responsive factor (*ERF*) family genes and potassium transporter family genes for RCA formation were the potential candidate genes involved under waterlogging conditions. Several genotypes, which performed consistently well under different conditions, can be used in breeding programs to develop waterlogging-tolerant varieties.

## 1. Introduction

Waterlogging is one of the prominent abiotic stresses due to climate change and one of the major constraints for barley production worldwide. Waterlogging occurs when the soil is inadequately drained, or deposited water cannot seep into it efficiently, producing the existing water fraction in the soil surface layer 20% higher than the field water capacity [1,2]. Approximately 16% of the fertile areas around the earth undergo regular waterlogging caused by excessive rainfall, lack of soil drainage, and irregular topography, which result in severe economic loss [3].

In plants, the availability of oxygen for respiration is hampered in waterlogged organs because gas diffusion in water is about 10,000 times slower than that in the air [4,5]. To continue the energy supply, it is necessary for the waterlogged organs to switch over to anaerobic mode for energy production [6]. However, the energy trade-off via glycolysis and fermentation is inefficient when waterlogging is prolonged [4,7]. To survive from long-term waterlogging events, aerobic respiration for plants must be maintained via oxygen transport to enhance internal oxygen diffusion [8]. Root morphological and anatomical traits contribute to plant adaptation to waterlogged soils. One of such morphological traits is the formation of adventitious roots (AR), a trait that exists in many different plant species [9,10,11,12,13]. New adventitious roots facilitate water and nutrient uptake under hypoxia stress [14]. The formation of AR minimizes the distance for oxygen diffusion and improves gas diffusivity [15]. AR usually originate from the waterlogged part of hypocotyls or basal stem region, and such adaptation can replace the deteriorating primary roots [15]. Therefore, the adaptive responses of AR development to waterlogging might be more important than those of primary roots for survival in waterlogged soil [16].

Root cortical aerenchyma (RCA) formation in adventitious roots is another feature that enables plant adaptation to waterlogging conditions [17,18] by enhancing the internal diffusion of atmospheric and photosynthetic oxygen from the aerial parts to the waterlogged roots, allowing the roots to maintain aerobic respiration [19]. Aerenchyma formation increases the porosity of roots above the usual levels promoted by intercellular spaces [20]. The increase in root porosity of tolerant genotypes in response to waterlogging stress could represent the adaptation to hypoxic or anaerobic conditions [21]. RCA is a developmental process that is triggered by the hormone ethylene. Its activity contributes to the opening of gas spaces within parenchymatic tissues due to programed cell death (PCD) and cell wall modifications [16]. The formation of RCA has been studied extensively in the roots of various plant species, including barley [9], rice [22], maize [23], zea [11], wheat [21], and sugarcane [24].

Waterlogging also causes a sharp decrease in soil redox potential, resulting in very significant changes to the soil elemental profile, and significant accumulation of toxic substances occurs in soil [25]. Materials potentially toxic to plants that are accumulated in waterlogged soils include reduced manganese, iron, hydrogen sulphide, various organic acids, and ethylene [26]. AR and RCA formation are most directly related to a plant’s superior ability to adapt to anoxic/toxic conditions [25]. Our previous study identified that those genotypes with higher proportion of AR and RCA formation provided substantial tolerance to prolonged waterlogging conditions and improved grain yield [18].

Genome-wide association studies (GWAS) have been applied in the trait-associated genetic studies with a large number of single nucleotide polymorphisms (SNPs) in many plant species, such as *Arabidopsis thaliana* [27], rice [28], and wheat [29,30]. Many QTL for waterlogging tolerance in barley have been detected [31,32,33], including those for aerenchyma formation [32,34], total root dry weight [35,36], root porosity [34], and ROS formation [37]. Most of these studies focused on only double haploid (DH) populations. GWAS is an alternative way for detecting QTL/MTA, which became an approach for unraveling the molecular genetic basis underlying the natural phenotypic variation [38]. GWAS methods have been developed to overcome many of the inherent drawbacks of the traditional QTL approaches due to a deep history of recombination events [39]. Through the recent developments in genome-wide studies, hundreds of accessions encompassing thousands of gene loci can be genotyped using high-throughput markers to enhance the efficiency of existing breeding approaches [40,41,42]. Thus, GWAS can specifically locate polymorphisms and the responsible genetic loci that are accountable for phenotypic variations to allow gene-targeted explorations [43,44].

The first GWAS for waterlogging tolerance in a wide-ranging barley collection under controlled field conditions has been conducted recently, targeting different agronomic traits and yield components [45], but did not investigate AR development and RCA formation. In this study, we used a much greater number of genotypes collected worldwide, which leads to more reproducible results [38], to (1) detect the novel MTA controlling natural phenotypic and genetic variation of AR and RCA formation in response to waterlogging; and (2) identify potential candidate genes for AR and RCA formation. Our main goal was to understand the key traits contributing to waterlogging tolerance in barley and to develop molecular markers that can be used in marker-assisted selection for accelerating a breeding program to enhance waterlogging tolerance in barley. Our secondary goal was to reproduce evidence for previous findings, using different growing conditions and a larger pool of genotypes.

## 2. Results

### 2.1. Waterlogging Tolerance of Barley Accessions

**Figure 1:**
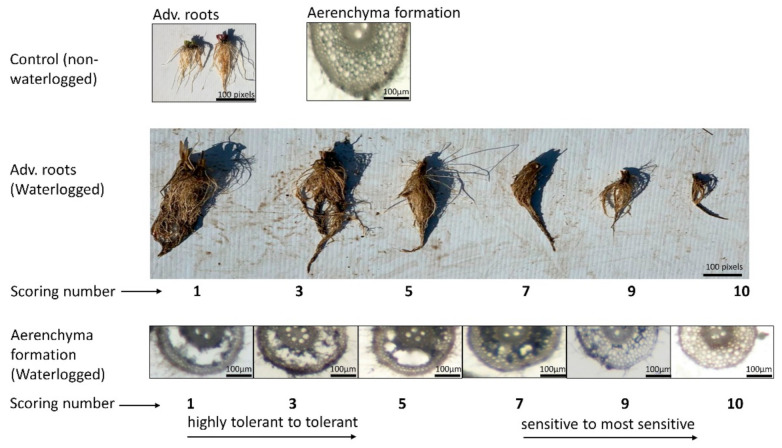
Adventitious root and aerenchyma formation under control and waterlogged conditions. Under control conditions, adventitious roots were not affected, and no aerenchyma was formed. However, under waterlogging conditions, adventitious roots were affected, and aerenchyma was formed. Waterlogging tolerance was divided into four levels: highly tolerant (score 1–3), tolerant (score above 3–5), sensitive (score above 5–7), and highly sensitive (score above 7–10).

**Figure 2:**
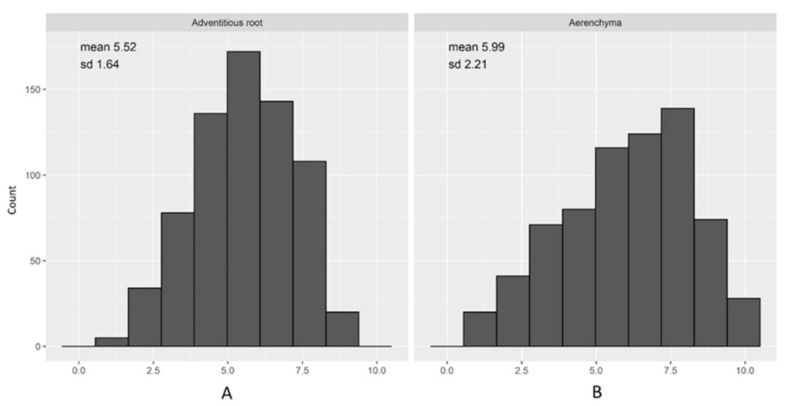
Frequency distribution of adventitious root development (**A**) and aerenchyma formation scores (**B**). The numbers are averages over all trials. A lower score means more tolerant.

The development of adventitious roots was quantified by allocating scores to plants (on a 1 to 10 scale) after sixty days waterlogging treatment when most of the sensitive lines showed prominent visual symptoms. The most tolerant varieties were scored 1 (a large number of AR and no dead roots), and the most sensitive/intolerant plants scored 10 (very few roots or all dead) (Figure 1). Based on these root scores, waterlogging tolerance was divided into four levels: highly tolerant (score 1–3), tolerant (score above 3–5), sensitive (score above 5–7), and highly sensitive (score above 7–10). Based on the average values from two tank and one field experiments, the AR scores show that our barley population consists of approximately 7% highly tolerant, 31% moderately tolerant, 40% sensitive, and 20% highly sensitive accessions (Figure 2A). For RCA formation, we also used a scoring number from 1 to 10, with the interpretations of scores shown in Figure 1; as for AR scores, lower values indicate higher tolerance to waterlogging. Among the RCA formation genotypes, about 14% genotypes were highly tolerant, 22% moderately tolerant, 27% sensitive, and 34% highly sensitive (Figure 2B). The frequency distributions, means of all the traits, and their standard deviation are presented in Figure 2A,B. Based on the combination of adventitious root development and aerenchyma formation, genotypes are clustered into four groups: (a) tolerant lines with a large amount of AR and a high proportion of RCA formation (both scores close to 1), (b) tolerant lines with a high number of AR but poor ability for RCA formation, (c) intolerant lines with a lower number of AR and a high proportion of RCA, and (d) intolerant lines with limited AR and limited proportion of RCA (both scores approaching 10) (Table S1). Overall, AR development and RCA formation were relatively consistent, with the correlation being r = 0.58 (*n* = 691). The broad-sense heritability [46] estimates (as a measure of repeatability, or “intraclass correlation”, of results for the same genotypes across experimental conditions) for AR development and RCA formation were 0.44 and 0.83, respectively.

### 2.2. SNP Distribution and Principal Components in Barley GWAS Population

SNPs are distributed all over the chromosomes (Figure S1), with more in the tail end of the chromosome. The frequency of the SNPs in the middle area is less mainly due to cross-overs in centromere area of the chromosome. Frequency of the SNPs of most of the chromosomes ranged from 0 to 25 within one million base (Mb) window size. The first and second principal components account for only 8.1% and 5.1% of the variance in the genotype data matrix (Figure S2).

### 2.3. Genome-Wide Association Study in Barley under Waterlogging Conditions

**Figure 3:**
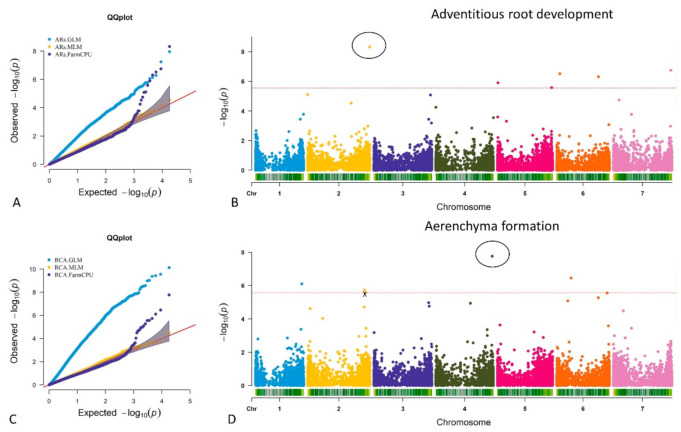
The quantile–quantile (QQ) plots from the models (GLM, MLM, and FarmCPU) are in (**A**,**C**); The FarmCPU model highlighted six potentially significant marker trait associations (MTA) for adventitious root formation from average scores of all the experiments (**B**); Five significant MTA were identified for root cortical aerenchyma (RCA) formation in adventitious roots (**D**). The highest −log_10_ (*p*) MTA for adventitious root and aerenchyma formation were approximately 9 and 8 on chromosome 2H and 4H, respectively ((**B**) (circle); (**D**) (circle)). Two MTA on 2H were located at similar positions (cross mark), thus counted as one MTA (**D**).

We first examined three different GWAS models: generalized linear models (GLM), mixed linear models with random effects (MLM), and FarmCPU models. The quantile–quantile (QQ) plots from these different models (GLM, MLM, and FarmCPU) are shown in Figure 3A,C. The GLM model produced many −log_10_ (*p*) values beyond what would be expected (i.e., there is an abundance of SNPs that appear relevant); the MLM method identified no significant SNPs; and the FarmCPU had a reasonably modest set of extreme –log_10_ (*p*) values. Accordingly, we focus on the SNPs identified from the FarmCPU modeling. 

#### 2.3.1. MTA for Adventitious Root Formation

**Figure 4:**
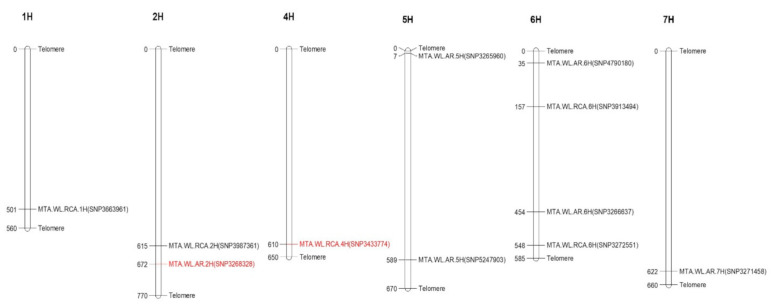
Genetic linkage map of identified MTA. MTA.WL.AR stands for the association between markers and AR development under waterlogging condition; MTA.WL.RCA stands for the association between markers and RCA formation under waterlogging condition. Red-color MTA are the lead MTA. Physical position of the chromosome was used to detect the respective MTA.

Using the FarmCPU model, six significant marker trait associations (MTA) were identified based on the average scores of adventitious roots (AR) formation (Figure 3B). Table S2 lists all significant MTA. These MTA are located on chromosomes 2H (SNP3268328), 5H (SNP3265960 and SNP5247903), 6H (SNP4790180 and SNP3266637), and 7H (SNP3271458) (Figure 4). The most significant MTA was on chromosome 2H with the observed −log_10_ (*p*) values of about 9, with the tolerant allele of the SNP3268328 increasing the tolerance score by 0.72 (Table S2). 

#### 2.3.2. MTA for Aerenchyma Formation

Five significant MTA were identified for RCA formation in adventitious roots (Figure 3D, Table S2). The MTA are on chromosomes 1H (SNP3663961), 2H (SNP3987361), 4H (SNP3433774), and 6H (SNP3913494 and SNP3272551) (Figure 4). SNP3433774 on 4H showed the most significant association with RCA, with an observed −log_10_ (*p*) value of about 8. The tolerant allele of SNP3433774 increased the average RCA score by 1.03 (Table S2). Two MTA on 2H were located at similar positions, thus counted as one MTA (SNP3987361) (Figure 3D). This MTA is in a close position to the MTA on 2H for AR development. Similarly, the MTA on 6H for RCA formation were also located at near position to the MTA for AR development on 6H (Figure 4).

#### 2.3.3. The Combined Effect of Different Alleles

**Figure 5:**
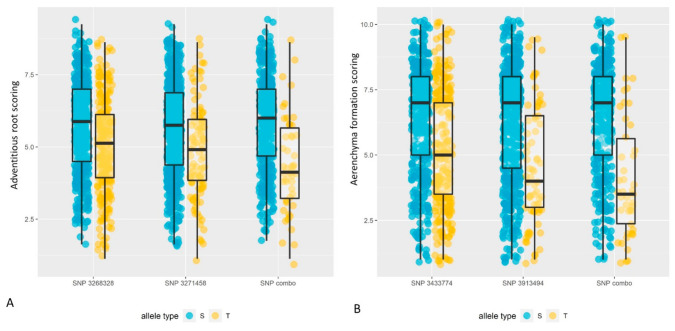
Allele effects of tolerant alleles (“T”) and susceptible alleles (“S”) from (**A**) AR development and (**B**) RCA formation. The data are for the top two SNPs (i.e., the most extreme *p*-values from the FarmCPU analysis) for AR development and RCA formation, respectively. The “SNP combo” data is from varieties, which have either both respective tolerant alleles, or both respective susceptible alleles. For AR development: *n* = 694 for SNP 3268328, *n* = 694 for 3271458, and *n* = 467 for the SNP combo. For RCA formation: *n* = 691 for SNP3433774, *n* = 691 for SNP3913494, and *n* = 466 for the SNP combo. The data are slightly jittered (at random) to expose the distributions, and the overlaying boxplots identify the interquartile range and the median.

Allelic effects of the two markers showing the most significant association (i.e., the most extreme *p*-values from the FarmCPU analysis) with average adventitious root development and aerenchyma formation scores are shown in Figure 5. Overall, tolerant alleles (“T” in the charts) showed lower visual scoring (healthier roots or greater proportion of RCA) than susceptible alleles. 

The two most significant MTA for AR development showed additive effects, in the sense that the average score of the genotypes with both tolerant alleles was much lower than those with only one tolerant allele (as evidenced by the “SNP combo” data in Figure 5A). Combined allelic effects of MTA for aerenchyma formation were also significant in the same sense, and a greater number of the genotypes with both tolerant alleles showed a greater proportion of RCA formation (as evidenced by the “SNP combo” data in Figure 5B). 

Simple *t*-tests comparing the means of varieties with the two most susceptible alleles (S) versus varieties with the two most tolerant alleles (T) were as follows. The “SNP combo” difference in means for adventitious root development scores (T minus S) was −1.38, with a 95% confidence interval of (−1.86, −0.89) and −log_10_ *p*-value of 7.46. The “SNP combo” difference in means for aerenchyma formation scores (T minus S) was −2.30, with a 95% confidence interval of (−2.94, −1.65) and −log_10_ *p*-value of 11.15. The rest of the test details are in Table S4.

### 2.4. Candidate Genes for Adventitious Root and Aerenchyma Formation

All six MTA for AR development and five MTA for RCA formation were considered as favorable MTA for candidate genes identification. A search for possible candidate genes for AR and RCA formation within the different regions of MTA was conducted. The putative domain family genes that were located within 2 Mb window from the MTA were considered as a possible candidate family gene. Waterlogging-responsive genes are located within the MTA region for AR, and RCA formations are listed in Table S3. 

There are 28, 36, 12, 19, and 42 candidate genes that are located within 2 Mb screen of *MTA.WL.AR.2H* (*SNP3268328*), *MTA.WL.AR.5H* (*SNP3265960* and *SNP5247903*), *MTA.WL.AR.6H* (*SNP4790180* and *SNP3266637*), and *MTA.WL.AR.7H* (*SNP3271458*), respectively. Among all candidate genes, some of the gene families are highly induced by waterlogging or the SNPs of the MTA located inside the genes are considered decent candidate family genes. The two gene families that are most closely associated with the markers are *MFS* transporter superfamilies (*HORVU5Hr1G093350* and *HORVU5Hr1G093390*) and leucine-rich repeat (*LRR*) family genes (*HORVU5Hr1G093410* and *HORVU7Hr1G107310*).

The highly hypoxia-stress-responsive family genes or those closely associated with most significant MTA were considered for possible candidate family genes for RCA formation. There are 31, 17, 12, and 27 candidate genes that are located within 2 Mb distance of *MTA.WL.RCA.1H* (*SNP3663961*), *MTA.WL.RCA.2H* (*SNP3987361*), *MTA.WL.RCA.4H* (*SNP3433774*), and *MTA.WL.RCA.6H* (*SNP3913494* and *SNP3272551*) region, respectively. Among them, *AP2*/*ERF* domain superfamily genes (*HORVU4Hr1G079630*) and potassium transporter family genes (*HORVU4Hr1G079150*) are the most likely candidate genes for RCA formation under waterlogging conditions.

## 3. Discussion

Barley waterlogging tolerance is influenced by many mechanisms, including RCA formation [17] and the development of AR [20]. In the past decade, many MTA/QTL have been mapped on all seven chromosomes of barley for waterlogging tolerance [31,32,33,37,47]. The value of MTA/QTL in breeding programs depends on their effects (and combined or additive effects) and whether they respond well in different backgrounds and environments. In this study, we identified some novel MTA. We also identified some other MTAs that are located at similar/near positions to those reported previously, which are associated with other traits. This element of “reproducibility” confirms the importance of the identified MTA/QTL [32]. In addition, the significant MTA for AR and RCA formation showed additive effects, thus showing the potential to be combined into more tolerant genotypes. 

### 3.1. Formation of ARs and RCA under Waterlogging 

Roots are the first organs to be affected by waterlogging, with the death of seminal roots being observed soon after hypoxia onset [48]. AR development is a typical responsive trait of waterlogged plants, which can partially replace the damaged seminal root and create more aerenchyma to improve the capability for internal oxygen transportation [49]. The formation of AR and RCA under waterlogging conditions has been reported for many crops, such as maize, soybean, tomato, and a deep-water rice [9,13,50,51,52]. Our previous experiments identified that AR and RCA formation are some of the most important traits associated with waterlogging tolerance in barley [9,18]. The introgression of a single major RCA-QTL into commercial barley varieties can increase the waterlogging tolerance without significant negative effects on yield and quality [18]. 

Major QTL for waterlogging-triggered adventitious roots have been reported in maize [53,54] and cucumber [55]. Aerenchyma formation in adventitious roots has been suggested as a reliable method for screening waterlogging tolerance in maize and barley [16,32,54]. In this experiment, a total of 11 MTA was identified for AR and RCA formation under waterlogging stress using the 697 barley accessions collected worldwide. The most significant MTA (*SNP3268328*) for AR development was identified on 2H, and the most significant MTA (*SNP3433774*) for RCA formation was on chromosome 4H (Figure 4). *MTA.AR* (*SNP3268328*) on 2H was within 10 Mb from barley waterlogging tolerance QTL identified from the Yerong/Franklin and Franklin/TX9425 populations [31,33]. *MTA.RCA* (*SNP3433774*) on 4H was within 2–5 Mb distance from the major QTL for RCA formation and waterlogging tolerance reported in several DH populations [9,32,33,48]. 

Apart from the lead MTA, we also identified some other significant MTA for AR and RCA formation. Some of them are located at near positions or inside the previously reported QTL for waterlogging tolerance. For example, *MTA.WL.RCA.1H* (*SNP3663961*) is positioned within 200 Mb from the QTL for plant survival (*QSur.yf.1H*) in the Yerong/Franklin population [56], and hypoxia shoot dry weight (*QHSDW.1H*) in the Franklin/YYXT population [34] under waterlogging conditions. *MTA.WL.RCA.2H* (*SNP3987361*) is found about 50 Mb from the MTA for barley spike per plant [45] and inside the QTL (*GSw1.1*) for waterlogging grains per spike in the Yerong/Franklin population [57]. *MTA.WL.AR.5H* (*SNP5247903*) is located within 2 Mb from MTA for spike per plant under waterlogging treatment [45], and inside the *QSlsw.YG.5H* for waterlogging and salinity tolerance in YSM1/Gairdner population [58], and *QWl.TxNn.5H* in TX9424/Naso Nijo for waterlogging tolerance [59]. *MTA.WL.AR.6H* (*SNP4790180*), *MTA.WL.AR.6H* (*SNP3266637*), *MTA.WL.RCA.6H* (*SNP3913494*), and *MTA.WL.RCA.6H* (*SNP3272551*) were situated inside the previously identified QTL for plant survival (*QSur.yf.6H*) and chlorophyll content (*QLC.yf.6H*) in Yerong/Franklin population [56]. We consider *MTA.WL.AR.5H* (*SNP3265960*) and *MTA.WL.AR.7H* (*SNP3271458*) for adventitious root formation were the novel MTA, as there was no identified QTL/MTA located near to those MTA under waterlogging conditions.

### 3.2. Candidate Genes

AR and RCA formation induced by waterlogging is a complex process that is regulated by multiple hormone signaling pathways. Ethylene, as the primary signal in response to waterlogging stress, acts in both developmental modes of adventitious rooting, promotes auxin transport, and increases the sensitivity to auxin in the rooting zones, thereby facilitating adventitious root development [60]. The production of adventitious roots is directly related to ethylene production. Ethylene has been implicated in signaling cell death in the formation of aerenchyma in the cortex of adventitious roots of maize subjected to hypoxia [61]. 

Genome-wide analysis on ethylene-responsive factors (*ERF*) family genes has found that *HvERF2.11* in barley [62], *AvERF73* and *AvERF 78* in *Actinidia valvata* [63], *AP2/ERF* family genes in maize [64] are highly induced by waterlogged conditions. The *AP2/ERF* family genes are coincided with *MTA.WL.RCA* in chromosomes 1H (*SNP3663961*) (*HORVU1Hr1G075040*, three members (mem)), 4H (*SNP3433774*) (*HORVU4Hr1G079630*, one mem); and 6H (*SNP3272551*) (*HORVU6Hr1G082820*, one mem), (*HORVU6Hr1G082840*, one mem) and (*HORVU6Hr1G082880*, five mem). We believe that *AP2/ERF* family genes might play a vital role in RCA formation in barley.

Potassium also plays an important role as a determinant of cell fate, with cytosolic K^+^ acting as a trigger of programed cell death under a range of biotic and abiotic stress conditions, and the plant’s ability to take up K^+^ is significantly reduced under O_2_- deficient conditions [25]. Under waterlogging conditions, the metabolism of gamma aminobutyric acid (GABA) can bind directly to the guard cell outward rectifying K^+^ channels, thereby improving hypoxia tolerance [25,65]. Potassium transporter family genes (*HORVU4Hr1G079150*, 11 mem) and (*HORVU7Hr1G107400*, 32 mem) are positioned within 1 Mb from *MTA.WL.RCA.4H* and *MTA.WL.AR.7H*, respectively. High-affinity nitrate transporters play an important role in nitrogen acquisition under anaerobic conditions and are the members of the major facilitator superfamily (*MFS*) transporter genes [66]. *MFS* family genes (*HORVU2Hr1G085160*, 10 mem) and (*HORVU2Hr1G085260*, 5 mem) are located within 1 Mb areas of *MTA.WL.RCA.2H* (*SNP3987361*), and (*HORVU5Hr1G093350*, 20 mem) and (*HORVU5Hr1G093390*, 4 mem) are also within 2 Mb of *MTA.WL.AR.5H* (*SNP5247903*). Leucine-rich repeat receptor (*LRR*)-like kinases play a fundamental role in sensing external signals and regulating gene expression responses at the cellular level and induced by abiotic stresses [67]. The responses of this gene increased the oxidative stress tolerance in the germination and early root growth in Arabidopsis [68]. One *LRR* gene (*HORVU5Hr1G093410*), six *LRR* genes (*HORVU6Hr1G033670*) and eight *LRR* genes (*HORVU7Hr1G107310*) are found within 1 Mb *MTA.WL.AR.5H* (*SNP5247903*), *MTA.WL.RCA.6H* (*SNP3913494*), and *MTA.WL.AR.7H* (*SNP3271458*), respectively.

Under waterlogging conditions, aerobic respiration is restricted, and anaerobic respiration (glycolysis and fermentation) for energy generation is an adaptive strategy of plants [7]. We also found a substantial number of genes related to glycolysis and fermentation. *FHY3/FAR1* family genes operate downstream of a metabolic pathway that converts the pyruvate derived from glycolysis into alanine. The *FHY3/FAR1* family genes (*HORVU5Hr1G092140*, one mem) are located within 2 Mb of *MTA.WL.AR.5H* (*SNP5247903*). Due to a deficiency of oxygen under waterlogging conditions, plants adjust mitochondrial respiration to glycolysis by fermentation of pyruvate to ethanol via pyruvate decarboxylase (PDC) [69]. Mitochondrial pyruvate carrier family genes (*HORVU2Hr1G085120*, two mem) are positioned within 1 Mb of *MTA.WL.RCA.2H* (*SNP3987361*). 

Root cap formation *miRNA* genes *miR164*, *miR167* and *miR393* are involved in lateral root development and plant cell detoxification by scavenging the reactive oxygen species, thus protecting the cellular structure from the damage induced under waterlogging [70]. Twenty-eight RNA-binding domain superfamily genes (*HORVU1Hr1G075000*) are located within 200 kb from *MTA.WL.RCA.1H* (*SNP3663961*). *MYB* genes are key factors in physiological processes, root formation, shoot growth, organ development, hormone signal transduction, metabolism, and responses to waterlogging stress [71]. Five *MYB* domain family genes (*HORVU6Hr1G066000*) are found within 200 kb region of *MTA.WL.AR.6H* (*SNP3266637*). Heat shock genes induced by waterlogging stress are involved in many cellular processes, including protein translocation across membranes and regulation of protein degradation [72]. Heat shock 70 kD peptide-binding domain family genes (*HORVU6Hr1G082510*, 10 mem) and (*HORVU6Hr1G082600*, 4 mem) are positioned within 1 Mb of *MTA.WL.RCA.6H* (*SNP3272551*) and (*HORVU7Hr1G107190*, 2 mem) in *MTA.WL.AR.7H* (*SNP3271458*). Aquaporin transporter family genes are involved in the transport of gases, end products of anaerobic respiration, and signaling molecules [73]. A total of 16 aquaporin transporter genes (*HORVU2Hr1G096360*) are located within 500 kb region of *MTA.WL.AR.2H* (*SNP3268328*). Haloacid dehalogenase-like hydrolase (*HAD*), acyl-coA synthase, and protein kinase regulatory super family genes are induced by hypoxia [74,75]. Ten *HAD* family genes (*HORVU5Hr1G003910*), two acyl-coA binding protein superfamily genes (*HORVU6Hr1G016280*) are found within 1 Mb region of *MTA.WL.AR.5H* (*SNP3265960*) and *MTA.WL.AR.6H* (*SNP4790180*), respectively. Protein kinase regulatory super family genes (*HORVU2Hr1G095950*, 2 mem, and *HORVU2Hr1G096350*, 10 mem), and (*HORVU6Hr1G016380*, 1 mem, and *HORVU6Hr1G066120*, 4 mem) are positioned within 1 Mb areas of *MTA.WL.AR.2H* (*SNP3268328*) and *MTA.WL.AR.6H* (*SNP4790180* and *SNP*3266637), respectively.

To conclude, we propose that ethylene responsive factor (*ERF*) family genes and potassium transporter family genes for RCA formation, and *MFS* transporter superfamily genes and leucine-rich repeat (*LRR*) family genes for AR development, could both be decent candidate genes that are involved in barley plant’s response to waterlogging conditions. The other potential candidate genes that have been reported to be associated with waterlogging tolerance might also play an important role for AR and RCA formation in barley.

## 4. Materials and Methods

### 4.1. Plant Materials

A total of 697 barley accessions, collected worldwide, were used in this study. Our pool of barley genotypes includes approximately 30% that originate from China, 5% from commercial Australian varieties, 7% from wild barley of mixed global origin, and the balance primarily from Europe. The split of genotypes between two-row and six-row barley is approximately 70% and 30%, respectively.

DNA was extracted from leaf tissue collected at the 2-leaf seedling stage from a single plant per accession and genotyped with DArTSeq (http://www.diversityarrays.com/dart-application-dartseq, accessed on 1 February 2022). Over 33,000 DArT and 31,000 SNP markers were scored. After removing those with the same scores or with greater distortion and/or greater proportion of missing data, a total of 18,132 markers with less than 5% missing data were used for marker-trait association study.

### 4.2. Growing Conditions, Treatment, and Phenotyping 

Experiment one (tank): The 697 genotypes were grown in tanks under natural conditions at Tasmanian Institute of Agriculture in the 2020 and 2021 growing seasons (Figures S3 and S4). Four seeds of each genotype were sown in a tank with 0.10 m row space and 0.06 m between genotypes with two replications.

Experiment two (field): A field experiment was performed in 2021 under field waterlogging conditions at the research station at Tasmanian Institute of Agriculture, Launceston, Tasmania (Figures S3 and S4). Fifteen seeds of each from 697 genotypes were sown in 0.06 m rows with 0.25 m row space with two replications. 

Waterlogging treatment was imposed at 2–3 leaf stage in all the above experiments and continued for two months. Root cortical aerenchyma formation in adventitious roots was checked 10 days after waterlogging. Aerenchyma formation in adventitious root was scored 1 to 10 (Figure 1). Adventitious root scoring was also scored 1 to 10 after terminating two-month waterlogging treatment (Figure 1). Redox potential (Eh) was measured with redox probe, according to the method in our previous report [18]. Mean soil Eh value was significantly lower in the waterlogged treatments (−406 mV) than that in the well-drained control (+212 mV), indicating highly saturated and hypoxic conditions under waterlogging. Mean monthly rainfall, maximum and minimum temperature were comparable between the tank experiments and field experiment (Figure S3).

### 4.3. Genome-Wide Association Study

A genome-wide association study (GWAS) was conducted using rMVP package of R software [76]. In the association study, the notional *p*-value threshold to suggest a significant marker trait association (MTA) was set to −log_10_ (*p*) ≥ 5.5 (which is based on a singular test Type I error rate of 0.05, a simple Bonferroni adjustment [77], and 18,132 SNPs). 

#### Model Used for GWAS Analysis

In this study, GLM, MLM, and fixed and random model circulating probability unification (FarmCPU, [78]) models were compared using adventitious root development and root cortical aerenchyma formation scoring from the 697 accessions and 18,132 SNPs. The best model was selected based on quantile–quantile (QQ) plots. The outcome variable in each model was the average of the respective AR or RCA values across the different trials.

The FarmCPU model was found to be the most suitable one, which had a small number of extreme *p*-values (see the QQ plots). The FarmCPU method iteratively performs marker tests with pseudo-quantitative trait nucleotides (QTNs) as covariates in a fixed-effect model and keeps optimizing pseudo-QTNs in a random-effect model, and this process continues until no new pseudo-QTNs are added [78]. The joint capabilities of removing confounding effects between testing markers and kinship, preventing model overfitting, and controlling false positives has made the FarmCPU method one of the most efficient methods in modern GWAS [79]. Within our implementation of the rMVP model, we used the following settings: default imputation, no principal components, “max Loop” was set to 10 (this exceeded what was required for our data), and the internal “FaST-LMM” method was used. Significant markers were visualized with a Manhattan plot, and important *p*-value distributions from all the GLM, MLM, and FarmCPU results (expected versus observed *p*-values on a −log_10_ scale) are shown with quantile–quantile (QQ) plots.

### 4.4. Evaluation of Allelic Effect of Waterlogging Tolerance

The allele at the locus of the detected SNP responsible for increasing waterlogging tolerance (lower visual scoring (healthier roots or greater proportion of RCA)) is referred to as a “positive allele (T)” while the allele associated with increasing waterlogging susceptibility is classified as a “negative allele (S)” (Figure 5). The relative “effects” of SNPs were determined by the difference between the scores of negative alleles and those of positive alleles (Table S2), based on the rMVP output for the FarmCPU model.

### 4.5. Candidate Gene Associated with Waterlogging Tolerance 

SNPs at the level of −log_10_ (*p*) ≥ 5.5 were considered highly significant and were used to identify candidate genes. We used the physical positions and sequence of significant SNPs to identify the annotation of the high-confidence (HC) candidate gene. Potential candidate genes around ±2 Mb areas of the significant SNPs were searched against BLAST search—*Hordeum_vulgare*—Ensembl Genomes 64 (gramene.org) with default parameters. The barley reference genome assembly (IBSC v2; [80]) was used to identify the position of the possible candidate genes. The sequences of potential candidate genes were also blasted on the database of Grain Genes Class Browser: Probe (usda.gov, accessed on 17 March 2022) searching for their functional annotation. Candidate genes with a possible connection to waterlogging tolerance were taken into consideration. 

## 5. Conclusions and Prospective Research

Waterlogging seriously affects barley growth. GWAS analysis identified six and five MTA in barley for adventitious root development and aerenchyma formation, respectively, under waterlogging stress. The lead MTA that we identified can be combined with other significant MTA in breeding programs for increasing the proportion of RCA and improving AR development to elevate the barley waterlogging tolerance. Genes from the *MFS* transporter superfamily and leucine-rich repeat (*LRR*) family could be suitable targets for improving adventitious root development. The *AP2/ERF* domain superfamily genes and potassium transporter family genes might be good candidate genes for aerenchyma formation in adventitious root under waterlogging conditions. Several genotypes, which consistently produce well-developed adventitious roots and aerenchyma under different conditions can be used in breeding programs to develop waterlogging-tolerant varieties. 

Further research should focus on the following aspects. An association mapping population can be developed with barley diverse germplasm lines. Further fine mapping and positional cloning of MTA/QTL approaches can be applied to isolate the genes of interest, since the trait–loci association can be mapped with more precision and with high resolution. Pyramiding of two traits via marker-assisted backcross strategy to introgress the favorable alleles into commercial cultivated varieties will help develop advanced waterlogging-tolerant cultivars.

## Data Availability

Not applicable.

## Supplementary Materials

The following supporting information can be downloaded at: https://www.mdpi.com/article/10.3390/ijms23063341/s1.
