# Supplementary material for: Genome-Wide Association Study Reveals Marker Trait Associations (MTA) for Waterlogging-Triggered Adventitious Roots and Aerenchyma Formation in Barley
Source: Int J Mol Sci. 2022 Mar 19;23(6):3341. doi: 10.3390/ijms23063341 (PMC8954902; doi:10.3390/ijms23063341)
Supplement: Supplementary file 1 [file ijms-23-03341-s001.zip › ijms-1650260-supplementary.pdf]

International Journal of
Molecular Sciences

Article

Genome-wide association study reveals marker trait associations (MTA) for waterlogging-triggered adventitious roots and aerenchyma formation in barley

S M Nuruzzaman Manik, Md Quamruzzaman, Chenchen Zhao, Peter Johnson, Ian Hunt, Sergey Shabala, Meixue Zhou*

Supplementary tables

Table S1: List of the varieties with waterlogging tolerance

a) Tolerant line with large amount of AR and a higher proportion of RCA formation	b) Tolerant lines with a high number of AR but poor ability for RCA formation	c) Intolerant lines with a less number of AR and a high proportion of RCA	d) Intolerant lines with limited AR and limited proportion of RCA formation
B1079<br>B689<br>B797<br>HOR 4052<br>Kikai Hadaka<br>Kinai 8<br>REKA 1<br>Ricardo<br>Rika*(Balder*(Binder*Opal))<br>Russian 81<br>Shanghai 7<br>SM081413<br>Spanish landrace 309d<br>TAM402762<br>TAM407227<br>Tayeh 9<br>USA 8330-1679<br>WA10997<br>YERONG<br>XZ-2<br>Yipingsilengdamai	ARIMONT<br>Atribut<br>B779<br>BVA-9<br>BVC-10<br>BVC-3<br>Corvette<br>HOR 13447<br>Noire Maroc<br>181P94/1/3/1/1/1-2<br>20 UM<br>200 SM1 Barley 3002<br>261B1<br>2B03-3604<br>309T1<br>327Z1<br>67 UM<br>97IWFBC 12	Chengchou 5<br>MAR-82-E1138<br>Nord<br>Vanessa<br>X30<br>XVB19<br>X97<br>Yan 95221<br>Zhepi 4	Aizao 3<br>B1118<br>B1121<br>BM9845-6<br>Bolron<br>Buloke<br>BVC-21<br>Franklin<br>HOR 8850<br>K-8755<br>Kaputar<br>Linus<br>Najo nijo<br>Russian 74<br>Sahara<br>SLB 45-40<br>Spanish landrace 352<br>SR442<br>Viskosa<br>Yangpi 1

YPSLDM<br>YRJAR<br>YYXT			zheda<br>96-696 AC-13-11

Here, genotypes are showing alphabetically in each group. Based on the combination of adventitious root development and aerenchyma formation, genotypes are clustered into four groups: (a) tolerant lines with a large amount of AR and a high proportion of RCA formation (both scores close to 1), (b) tolerant lines with a high number of AR but poor ability for RCA formation, c) intolerant lines with a less number of AR and a high proportion of RCA and (d) intolerant lines with limited AR and limited proportion of RCA (both scores approaching 10).

Table S2: Significant SNPs from the traits adventitious root formation and aerenchyma formation under waterlogging conditions. Output is from the Farm CPU model within rMVP.

SNPs	Chr	Pos	Effect	SE	*p-value*	MTA	Traits
3663961	1	501,154,892	-1.58	0.35	7.95E-07	MTA.WL.RCA.1H	Aerenchyma
3987361	2	615,452,349	1.1	0.25	1.84E-06	MTA.WL.RCA.2H	Aerenchyma
**3268328**	**2**	**672,040,513**	**0.72**	**0.13**	**4.77E-09**	**MTA.WL.AR.2H**	**Adv. root**
**3433774**	**4**	**610,891,133**	**1.03**	**0.2**	**1.74E-08**	**MTA.WL.RCA.4H**	**Aerenchyma**
3265960	5	7,397,268	-0.49	0.12	1.26E-06	MTA.WL.AR.5H	Adv. root
5247903	5	589,500,162	0.48	0.11	2.68E-06	MTA.WL.AR.5H	Adv. root
4790180	6	35,557,165	0.62	0.13	3.05E-07	MTA.WL.AR.6H	Adv. root
3913494	6	157,415,479	1.5	0.31	3.49E-07	MTA.WL.RCA.6H	Aerenchyma
3266637	6	454,298,579	-0.57	0.12	4.94E-07	MTA.WL.AR.6H	Adv. root
3272551	6	548,336242	-0.65	0.16	2.81E-06	MTA.WL.RCA.6H	Aerenchyma
3271458	7	622,004,991	-0.86	0.18	1.81E-07	MTA.WL.AR.7H	Adv. root

Note: RCA= root cortical aerenchyma, AR=adventitious roots, Chr=chromosome, Pos=position; highlighted SNPs are the lead SNPs.

Table S3: Chromosomes, identified MTAs, traits, gene probe with position, and candidate genes family with number of genes in each family inside the bracket

	Gene probe	Start	End	Candidate genes
**Chr 1H**				
**3663961 (RCA)**	HORVU1Hr1G075000	509,572191	509,576703	RNA-binding domain superfamily (28)
	HORVU1Hr1G075040	509,584938	509,587493	AP2/ERF domain superfamily (3)
**Chr 2H**				
**3987361 (RCA)**	HORVU2Hr1G085120	615,297889	615,300905	Mitochondrial pyruvate carrier (2)
	HORVU2Hr1G085160	615,601770	615,606669	MFS transporter superfamily (10)
	HORVU2Hr1G085260	616,726973	616,730834	MFS transporter superfamily (5)
**3268328 (AR)**	HORVU2Hr1G095950	672,302733	672,303606	Protein kinase-like domain superfamily (2)
	HORVU2Hr1G096350	674,144117	674,153272	Protein kinase-like domain superfamily (10)
	HORVU2Hr1G096360	674,156049	674,158319	Aquaporin transporter (16)
**Chr 4H**				
**3433774 (RCA)**	HORVU4Hr1G079150	611,346020	611,350289	Potassium transporter (11)
	HORVU4Hr1G079630	613,053925	613,054630	AP2/ERF domain superfamily (1)
**Chr 5H**				
**3265960 (AR)**	HORVU5Hr1G003910	7,397268	7,479956	HAD superfamily protein (10)
**5247903 (AR)**	HORVU5Hr1G092140	587,616646	587,617430	FHY3/FAR1 family (1)
	HORVU5Hr1G093350	591,497184	591,501786	MFS transporter superfamily (20)
	HORVU5Hr1G093390	591,633650	591,639220	MFS transporter superfamily (4)
	HORVU5Hr1G093410	591,713561	591,715345	Leucine-rich repeat domain superfamily (1)
**Chr 6H**				
**4790180 (AR)**	HORVU6Hr1G016280	36,393078	36,393505	Acyl-CoA binding protein superfamily (2)
	HORVU6Hr1G016380	36882844	36884841	Protein kinase-like domain superfamily (1)
**3913494 (RCA)**	HORVU6Hr1G033670	155,477941	155,487662	Leucine-rich repeat domain superfamily (6)
**3266637 (AR)**	HORVU6Hr1G066000	455,535975	455,539510	Myb domain (5)
	HORVU6Hr1G066120	456,650915	456,654778	Protein kinase-like domain superfamily (4)
**3272551 (RCA)**	HORVU6Hr1G082510	548,585389	548,594631	Heat shock protein 70kD, peptide-binding dom. (10)
	HORVU6Hr1G082600	548,778315	548,780586	Heat shock protein 70kD, peptide-binding dom. (4)
	HORVU6Hr1G082820	549,583705	549,589117	AP2/ERF domain superfamily (1)
	HORVU6Hr1G082840	549,672098	549,674625	AP2/ERF domain superfamily (1)
	HORVU6Hr1G082880	549,919842	549,923137	AP2/ERF domain superfamily (5)
**Chr 7H**				
**3271458 (AR)**	HORVU7Hr1G107190	622,233404	622,235106	Heat shock protein 70 family (2)
	HORVU7Hr1G107310	622,587535	622,594179	Leucine-rich repeat domain superfamily (8)
	HORVU7Hr1G107400	623,072868	623,082441	Potassium transporter (32)

Table S4: T-test results for allele effects of tolerant allele ("T") versus susceptible allele ("S") from adventitious root development and aerenchyma formation. The data are for the top two SNPs (i.e. the most extreme p-values from the Farm CPU analysis) for adventitious root development and aerenchyma formation, respectively. The "SNP combo" data is from varieties which have either both respective tolerant alleles, or both respective sensitive alleles. The tests are simple t-tests for a difference in means (so the p-values differ from the more complex Farm CPU model). The base data for the tests is displayed in Figure 5.

Adventitious root t-test results for differences between T and S ('T minus S')

	SNP 3268328	SNP 3271458	SNP combo
Mean of tolerant (T)	5.03	4.86	4.43
Mean of susceptible (S)	5.75	5.62	5.80
Difference in means	-0.72	-0.76	-1.38
95% confidence interval for difference	(-0.97,-0.46)	(-1.11,-0.41)	(-1.86,-0.89)
Degrees of freedom	694	694	467
**-log10 *p*-value**	**7.23**	**4.63**	**7.46**

Aerenchyma t-test results for differences between T and S ('T minus S')

	SNP 3913494	SNP 3433774	SNP combo
Mean of tolerant (T)	4.59	5.25	4.15
Mean of susceptible (S)	6.14	6.38	6.44
Difference in means	-1.55	-1.13	-2.30
95% confidence interval for difference	(-2.09,-1.01)	(-1.46,-0.79)	(-2.94,-1.65)
Degrees of freedom	691	691	466
**-log10 *p*-value**	**7.64**	**10.12**	**11.15**

Supplementary figures

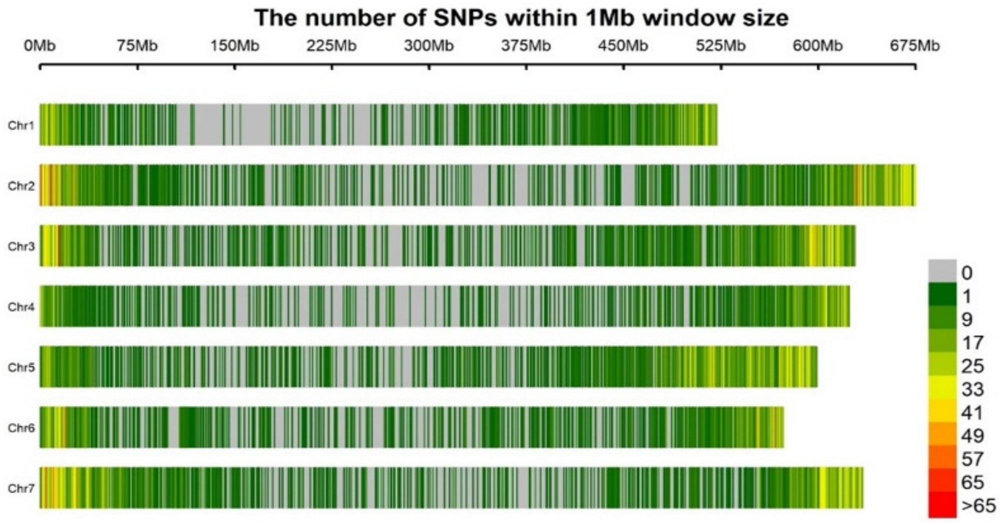

Figure S1: Total SNP distribution within the chromosome

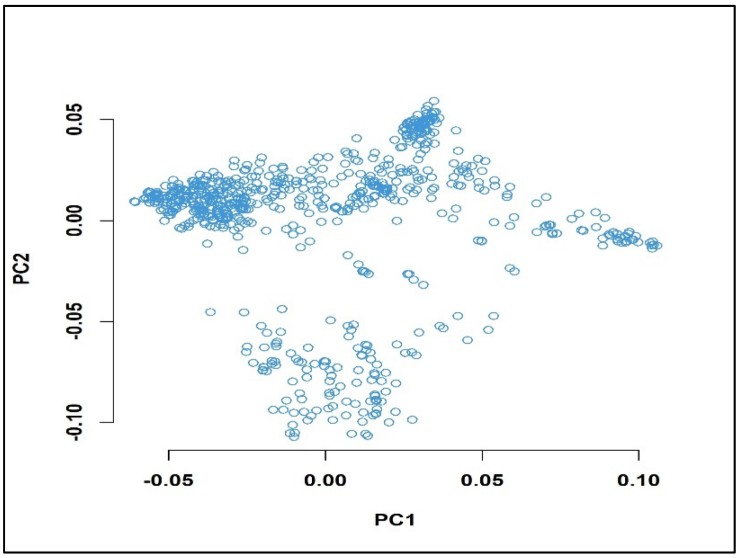

Figure S2: Principal component analysis

	Jan	Feb	Mar	Apr	May	Jun	Jul	Aug	Sep	Oct	Nov	Dec
**Rainfall**												
2020	40.7	61.1	101.8	140.6	52.9	26.1	23.2	87	54.6	110	40.1	91.2
2021	49	50.9	24	24.6	55.4	67.5	114.7	80.8	39.8			
**Monthly maximum temp**												
2020	25.3	24	21.5	17.6	15.4	13.1	13.3	13.9	15.9	17.8	22.8	21.8
2021	24.4	24	22.6	19.5	16.1	14	13	14.1	16			
**Monthly minimum temp**												
2020	13	12.3	10	8.9	5.7	3.2	1.3	3.7	6	8.2	10.6	10.1
2021	12.3	11.9	10.1	6.4	3.9	5	3.6	4.7	5.4			

Station:Launceston (Ti Tree Bend)
Number: 91237
Lat: 41.42° S
Lon: 147.12° E
Elevation: 5 m

EXP 01 in TANK | EXP 02 in TANK | EXP 01 in FIELD

Figure S3: Monthly rainfall, monthly maximum temperature, and minimum temperature in tank experiments and field experiment in 2020 and 2021

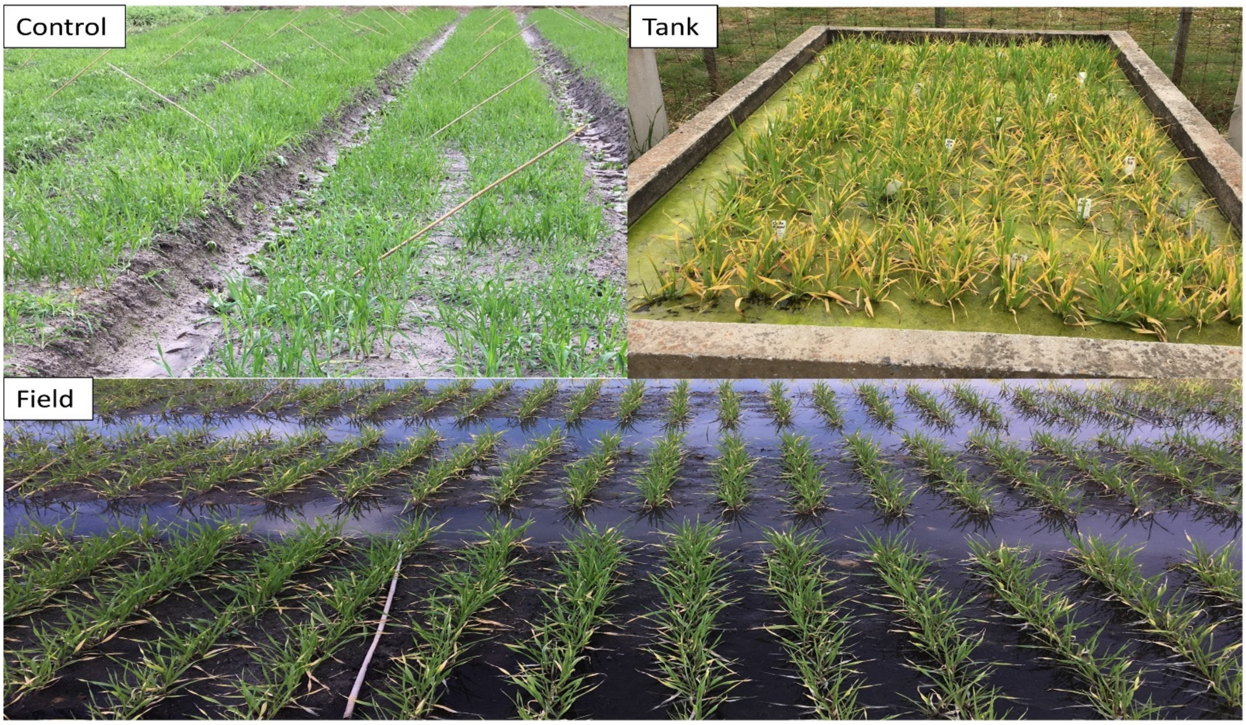

Figure S4: Photos of different experiments; control (non waterlogged field)**;** waterlogged tank trial**;** waterlogged field trial
